# Influence of laparoscopic surgery for endometriosis and its recurrence on perinatal outcomes

**DOI:** 10.1002/rmb2.12456

**Published:** 2022-04-06

**Authors:** Yosuke Ono, Kyoko Furumura, Osamu Yoshino, Hajime Ota, Yasushi Sasaki, Takao Hidaka, Yoshiyuki Fukushi, Shuji Hirata, Hideto Yamada, Shinichiro Wada

**Affiliations:** ^1^ Department of Obstetrics and Gynecology Teine Keijinkai Hospital Sapparo Japan; ^2^ 38529 Department of Obstetrics and Gynecology Tonami General Hospital Toyama Japan; ^3^ 38146 Department of Obstetrics and Gynecology University of Yamanashi Yamanashi Japan; ^4^ 41677 Department of Obstetrics and Gynecology Kurobe City Hospital Kurobe Japan; ^5^ Center for Recurrent Pregnancy Loss Teine Keijinkai Hospital Sapporo Japan

**Keywords:** endometriosis, laparoscopic surgery, perinatal outcome, placenta previa, pregnancy

## Abstract

**Purpose:**

It is unknown whether surgery for endometriosis or recurrence of endometriosis affects obstetric outcomes.

**Methods:**

A total of 208 pregnant women with a history of endometriosis were analyzed. Patients who had endometriomas >3 cm and no history of laparoscopic surgery for endometriosis were defined as non‐surgery group (*n* = 60), while those who had a history of surgery for endometriosis (*n* = 148) were defined as surgery group. We investigated the obstetric outcomes in 208 patients according to with or without postoperative recurrence of endometriosis and the time from surgery to pregnancy.

**Results:**

Among 177 cases of on‐going pregnancy, in surgery group, there were lower prevalence of placenta previa compared with non‐surgery group (8.5% vs. 23.4%; *p* = 0.020). Subgroup analysis revealed a decreased prevalence of placenta previa in postoperative non‐recurrence group (6.0%: *p* = 0.007) compared with non‐surgery (23.4%) and postoperative recurrence group (28.6%). Placenta previa was more prevalent in the patients who got pregnant more than 2 years after surgery (20.0%) than the patients who got pregnant within 2 years (2.4%: *p* = 0.002). Multivariate analysis revealed that the surgery was associated with a reduction in placenta previa (OR: 0.32, 95% CI [0.11–0.90]; *p* = 0.032).

**Conclusions:**

Pregnancy within two years after laparoscopic surgery for endometriosis may reduce placenta previa.

## Graphical abstract

We conducted a retrospective study to investigate the impact of laparoscopic surgery for endometriosis and postoperative recurrence of endometriosis on subsequent pregnancy outcomes. For severe endometriosis patients (revised American Society for Reproductive Medicine classification; stage III or IV), we found that the surgery for endometriosis significantly decreased the prevalence of placenta previa, but it increased again when the time between surgery and pregnancy was more than 2 years.

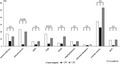

## 1 INTRODUCTION

Endometriosis is an estrogen‐dependent chronic disease associated with dysmenorrhea, pelvic pain, dyspareunia, and subfertility, and it affects approximately 10%–15% of reproductive‐aged women and impairs their quality of life.^1^, ^2^, ^3^, ^4^ The presence of endometriosis is also known to have an impact on pregnant women.^5^, ^6^, ^7^ There is increasing evidence of the association between endometriosis and pregnancy complications, such as miscarriage, preterm birth, placenta previa, hypertensive disorders of pregnancy (HDP), small‐for‐gestational‐age (SGA), and placental abruption.^8^, ^9^, ^10^, ^11^, ^12^, ^13^ Although surgical interventions for endometriosis, such as laparoscopic cystectomy, excision, ablation, and adhesiolysis, are performed when conservative treatments are ineffective, the influence of surgery on perinatal outcomes is still not fully understood.^14^ Several studies have examined the effect of surgery for endometriosis on perinatal prognosis. Innie et al.^15^ reported that women who underwent surgical treatment for endometriosis before pregnancy were found to have an elevated risk of placenta previa compared with those without endometriosis. Lin et al.^16^ reported that pregnant women with surgically diagnosed endometriosis have a higher risk for placenta previa, preterm delivery, fetal growth restriction (FGR), and cesarean delivery, even after adjustment for the impact of assisted reproductive technology (ART), compared to patients with normal pregnancy. These reports all compared obstetric complications between patients who underwent surgery for endometriosis before pregnancy and pregnant patients without endometriosis. However, so far, few studies have compared the perinatal prognosis between those who have endometriosis during pregnancy and those who have undergone surgery for endometriosis before pregnancy.

In this study, we retrospectively investigated whether a history of surgical treatment for endometriosis affects perinatal outcomes by comparing pregnant women with endometriosis, and no history of surgical treatment to pregnant women who underwent surgery for endometriosis before pregnancy.

## 2 METHODS

The Institutional Review Board of three institutions approved this study (2‐020039‐01; Teine keijinkai Hospital, No. 2020046; Tonami General Hospital, and No. 37, 2021; Kurobe City Hospital), which were conducted in accordance with the principles of the Declaration of Helsinki. The requirement for obtaining written informed consent was waived because of the retrospective nature of the study. This retrospective study was carried out to evaluate the perinatal outcomes of pregnant patients with endometriosis between January 1, 2005, and December 31, 2019, in three institutions. This study was conducted by collecting as much data as possible from 15 years ago, when endoscopic surgery for endometriosis could be performed stably at the research facilities. Endometriosis was diagnosed by pathological examination, transvaginal ultrasonography, and/or magnetic resonance imaging. Pregnant patients with current endometriosis or a history of endometriosis were included. Few previous studies have defined the severity of endometriosis and examined the perinatal prognosis. A recent review shows that severe endometriosis and non‐severe endometriosis have different effects on the obstetric outcome such as placenta previa.^17^ Therefore, in this study, patients with severe endometriosis, which is easily diagnosed by imaging studies, were included in the study. And ovarian endometrioma was defined as having >3 cm diameter, according to the highest stage of ovarian lesions in the revised American Society for Reproductive Medicine (re‐ASRM) classification. In the present study, we focused on how surgery for endometriosis affects the obstetric complications; therefore, we set the group that did not undergo surgery as the control group. The non‐surgery group comprised pregnant patients with ovarian endometrioma at the time a gestational sac was confirmed by transvaginal ultrasound. The surgery group included pregnant patients who had a history of laparoscopic surgery for endometriosis, with or without recurrence during pregnancy. Surgical treatments, such as laparoscopic cystectomy, excision, ablation, and/or adhesiolysis, were performed at any of the three institutions.

We investigated the perinatal prognoses of the two groups. In addition, in subgroup analysis, we compared the perinatal outcomes of the non‐recurrent group, in which the absence of recurrence of endometrioma was confirmed by transvaginal ultrasound in early pregnancy, and the recurrent group, in which endometrioma was present in early pregnancy. The recurrence of endometriosis was also defined as having an endometrioma >3 cm in diameter.

In general, a flare‐up of endometriosis symptoms within 2 years postoperatively is reported in 50% of patients with endometriosis^18^ and in 40%–80% of patients with endometriosis according to the American College of Obstetricians and Gynecologists guidelines.^19^ Therefore, we divided the surgery group into pregnant women for whom the time from surgery to pregnancy was within 2 years (<2Y group) and more than 2 years (>2Y group), regardless of the sign of the recurrence, and we compared the perinatal outcomes of the <2Y and >2Y groups. The patients’ clinical data were collected from the electronic medical records. Maternal characteristics included maternal age, body mass index before pregnancy, parity, the prevalence of leiomyoma, unilateral or bilateral of ovarian endometrioma, and the history of surgery for uterus and ART. ART included in vitro fertilization and intracytoplasmic sperm injection. Maternal outcomes included miscarriage (delivery before 22 gestational weeks), preterm labor (<37 gestational weeks), placenta previa, HDP, FGR, gestational diabetes mellitus (GDM), oligohydramnios, placental abruption, delivery mode, and the amount of blood loss at delivery. Neonatal outcomes included gestational age, birth weight, SGA, umbilical artery pH, and Apgar scores at 1 and 5 min. We excluded women with multiple pregnancies, congenital abnormalities, chronic hypertension or diabetes mellitus, endocrine diseases, cardiovascular diseases, and other internal complications.

The clinical data were analyzed using JMP version 10 (SAS Institute Inc.). In the comparison between the two groups, statistical analysis was performed using the Mann–Whitney U test, Fisher's exact test, and chi‐square test. For the comparison between the three groups, the analysis was performed by using the ANOVA test and the chi‐square test by m × n contingency table. The statistical significance was set at *p* < 0.05.

## 3 RESULTS

### 3.1 Non‐surgery group vs. surgery group

A total of 208 pregnant women with endometriosis were enrolled in this study. The non‐surgery group included 60 pregnancies with ovarian endometrioma and no history of surgical treatment, and the surgery group included 148 pregnancies after laparoscopic surgery for endometriosis. Maternal characteristics are shown in Table 1. There were no differences in age, body mass index, parity, and ART pregnancy rate, the percentage of bilateral ovarian endometrioma, leiomyoma, and the history of surgery for uterus between the two groups. In the non‐surgery group, the ovarian endometrioma diameter was 45.5 ± 16.4 mm (mean ± standard deviation) in the early pregnancy period. In the surgery group, the preoperative ovarian endometrioma diameter was 47.3 ± 15.5 mm, and the re‐ASRM score was 57.8 ± 61.6 points. The mean period from surgery to subsequent pregnancy was 29.3 ± 30.4 months.

**TABLE 1:** Maternal characteristics and perinatal outcomes of patients with endometriosis between the non‐surgery and surgery group*s*

Pregnant cases with endometriosis (*N* = 208)	Non‐surgery (*N* = 60)	Surgery (*N* = 148)	*p*‐Value
Age (years)	32.8 ± 4.9	33.2 ± 4.6	0.875
Body mass index (kg/m^2^)	21.6 ± 3.4	21.7 ± 3.3	0.654
Assisted reproductive technology	46.7% (28/60)	45.1% (64/142)	0.995
Parity	0.3 ± 0.6	0.4 ± 0.6	0.687
Revised‐American Society for Reproductive Medicine (points)	‐	57.8 ± 61.6	‐
Diameter of ovarian endometrioma (mm)	45.5 ± 16.4	47.3 ± 15.5	0.301
Period from surgery to pregnancy (month)	‐	29.3 ± 30.4	‐
Bilateral ovarian endometrioma	25.0% (15/60)	31.8% (47/148)	0.469
Leiomyoma	15.0% (9/60)	21.6% (32/148)	0.277
History of surgery for uterus	11.7% (7/60)	28.4% (42/148)	0.288
Miscarriage	21.7% (13/60)	12.2% (18/148)	0.089

Pregnant cases with endometriosis (*N* = 177) after 22 weeks of gestation	Non‐surgery (*N* = 47)	Surgery (*N* = 130)	*p*‐Value
Preterm delivery	17.0% (8/47)	9.2% (12/130)	0.179
Placenta previa	23.4% (11/47)	8.5% (11/130)	0.020
Hypertensive disorders of pregnancy	6.4% (3/47)	3.8% (5/130)	0.439
Fetal growth restriction	17.0% (8/47)	6.2% (8/130)	0.074
Gestational diabetes mellitus	4.3% (2/47)	6.9% (9/130)	0.730
Oligohydramnios	6.4% (3/47)	3.8% (5/130)	1.000
Placental abruption	2.1% (1/47)	0.8% (1/130)	0.466
Rupture of endometrioma during pregnancy	2.1% (1/47)	0% (0/130)	‐
Cesarean section	34.0% (16/47)	35.4% (46/130)	0.723
Gestational age at delivery (weeks)	38.3 ± 2.2	38.6 ± 3.2	0.285
Birth weight (g)	2858.8 ± 537.4	2887.9 ± 381.3	0.996
Small for gestational age	8.5% (4/47)	2.4% (6/130)	0.701
Umbilical artery pH	7.26 ± 0.06	7.28 ± 0.07	0.236
The blood loss at delivery (g)	845.7 ± 587.4	807.1 ± 543.6	0.871
Apgar score (1 min)	8.2 ± 0.5	8.3 ± 0.8	0.300
Apgar score (5 min)	9.1 ± 0.3	9.1 ± 0.5	0.507

Note

Data are presented as mean ± standard deviation or as *n* (%). *p*‐values < 0.05 are considered statistically significant.

The miscarriage rate tended to be lower in the surgery group than in the non‐surgery group (surgery group vs. non‐surgery group: 12.2%, 18/148 vs. 21.7%, 13/60, respectively; *p = *0.089) (Table 1). Among 177 cases of on‐going pregnancy, the surgery group exhibited a significantly lower prevalence of placenta previa compared with the non‐surgery group (8.5%, 11/130 vs. 23.4%, 11/47, respectively; *p = *0.020) and a lower tendency of FGR (6.2%, 8/130 vs. 17.0%, 8/47, respectively; *p = *0.074). The prevalence of preterm delivery, HDP, GDM, oligohydramnios, placental abruption, cesarean delivery, and SGA did not differ between the groups (Table 1). There was one case of apparent rupture of endometrioma during pregnancy in the non‐surgery group, but it did not result in emergent surgery. The rupture case of endometrioma was not confirmed in the surgery group. There were no differences in the mean gestational age, birth weight, umbilical artery pH, amount of blood loss at delivery, and Apgar scores at 1 and 5 min between both groups (Table 1).

### 3.2 Surgery group: Non‐recurrence vs. recurrence

**FIGURE 1:**
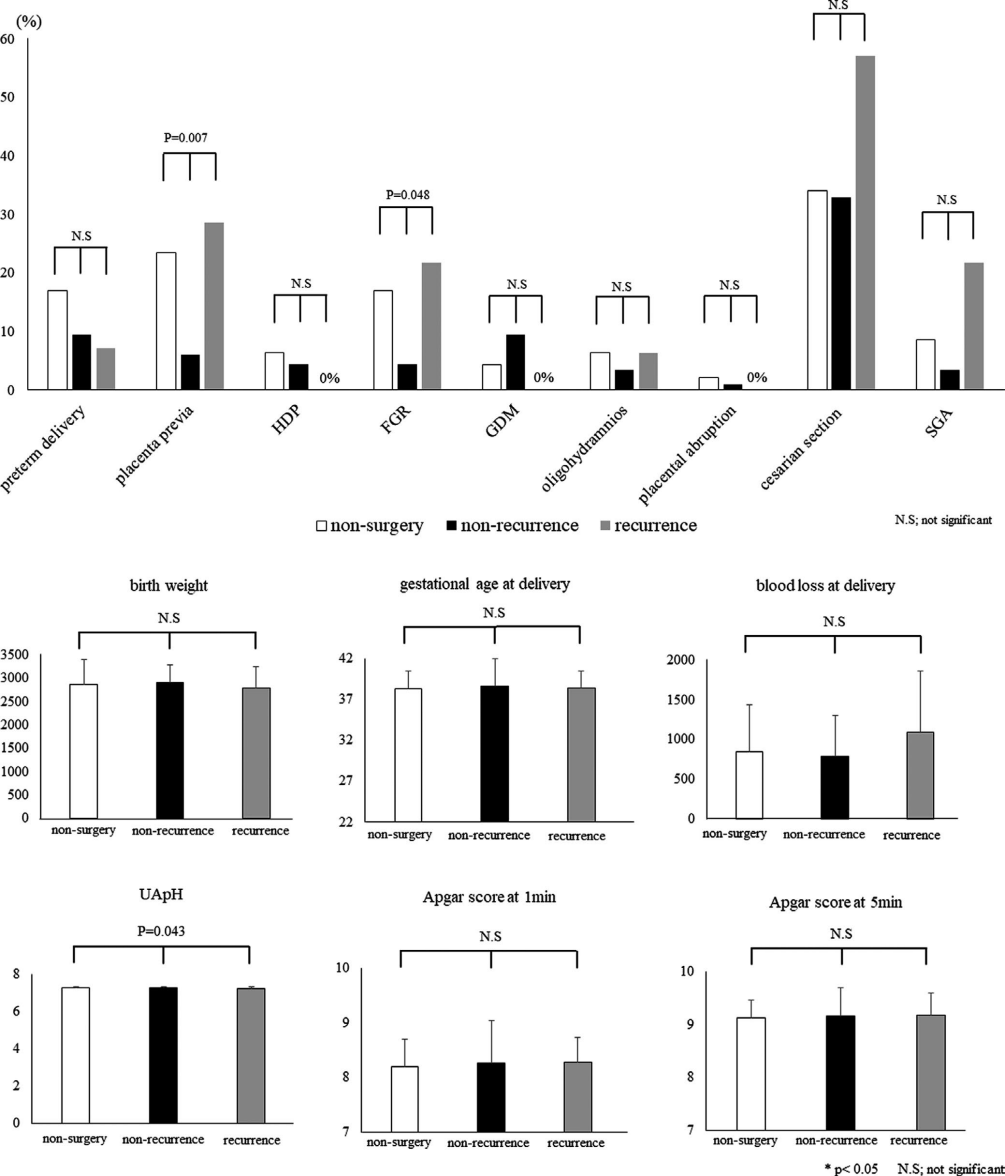
(A and B) Bar graphs illustrating perinatal outcomes in pregnant patients with endometriosis and no surgery, with postoperative non‐recurrence of endometrioma, and with postoperative recurrence of endometrioma Data are presented as mean ± standard deviation or as *n* (%). FGR, fetal growth restriction; GDM, gestational diabetes mellitus; HDP, hypertensive disorders of pregnancy; N.S., not significant; SGA, small for gestational age. *p*‐values < 0.05 are considered statistically significant

To examine the influence of recurrent endometrioma on perinatal prognosis, we sub‐categorized the surgery group into those with postoperative non‐recurrence of endometrioma >3 cm and those with postoperative recurrence. There were no differences in patient background between the non‐surgery, postoperative non‐recurrence, and postoperative recurrence groups (Table 2). There was no significant difference in the mean preoperative ovarian endometrioma diameter, the mean re‐ASRM score, and the mean period from surgery to subsequent pregnancy, miscarriage, and the percentage of bilateral ovarian endometrioma between the non‐recurrence and recurrence groups. The prevalence of leiomyoma tended to be lower in the postoperative recurrence group (*p = *0.091), and the percentage of surgical history for uterus tended to be higher in the postoperative recurrence group among the three groups (*p = *0.051) (Table 2). The prevalence of placenta previa and FGR was lower in the non‐recurrence group among the three groups (placenta previa; non‐surgery vs. non‐recurrence vs. recurrence: 23.4%, 11/47 vs. 6.0%, 7/116 vs. 28.6%, 4/14: *p = *0.007, FGR; 17.0%, 8/47 vs. 4.3%, 5/116 vs. 21.7%, 3/14: *p = *0.048) (Figure 1A, Supplemental Data 1). There were no differences in the other perinatal outcomes among the three groups (Figure 1B, Supplemental Data 1).

**TABLE 2:** Maternal characteristics between pregnant patients with endometriosis and no surgery, with postoperative non‐recurrence of endometrioma, or with postoperative recurrence of endometrioma

Pregnant cases with endometriosis (*N* = 208)	Non‐surgery (*N* = 60)^※1^	Postoperative non‐recurrence (*N* = 132)^※2^	Postoperative recurrence (*N* = 16)^※3^	*p*‐Value ^※1 vs. ※2 vs. ※3^
Age (years)	32.8 ± 4.9	33.3 ± 4.6	32.1 ± 4.3	0.632
Body mass index (kg/m^2^)	21.6 ± 3.4	21.9 ± 3.4	21.5 ± 2.2	0.731
Assisted reproductive technology	46.7% (28/60)	45.5% (60/132)	56.3% (9/14)	0.568
Parity	0.3 ± 0.6	0.4 ± 0.6	0.3 ± 0.5	0.880
Revised‐American Society for Reproductive Medicine (points)	‐	51.5 ± 32.9	54.5 ± 28.3	0.163^※2 vs. ※3^
Diameter of ovarian endometrioma (mm)	45.5 ± 16.4	48.2 ± 16.0	42.8 ± 12.2	0.409
Period from surgery to pregnancy (month)	‐	28.2 ± 29.4	39.0 ± 37.8	0.234
Bilateral ovarian endometrioma	25.0% (15/60)	31.8% (42/132)	31.3% (5/16)	0.627
Leiomyoma	15.0% (9/60)	24.2% (32/132)	0% (0/16)	0.091
History of surgery for uterus	11.7% (7/60)	28.0% (37/132)	12.5% (2/16)	0.051
Miscarriage	21.7% (14/60)	12.1% (16/132)	14.3% (2/14)	0.202

Note

Data are presented as mean ± standard deviation or as % (n/N). Statistical analysis was performed using the Mann‐Whitney U test, Fisher’s exact test, and chi‐square test. *P*‐values < 0.05 are considered statistically significant. ※1; Non‐surgery group, ※2; Postoperative non‐recurrence group, ※3; Postoperative recurrence group.

### 3.3 Surgery group: <2 years vs. >2 years after surgery

To investigate the influence of not only ovarian endometrioma but also endometriosis recurrence as a whole on perinatal outcomes, we stratified the surgery group into the <2Y and >2Y groups. We compared the perinatal outcomes of the non‐surgery group, the <2Y group, and the >2Y group.

There rate of ART tended to be higher in <2Y group (*p = *0.059) and the percentage of the surgical history for uterus tended to be higher in >2Y group (*p = *0.062) among the three groups.

**FIGURE 2:**
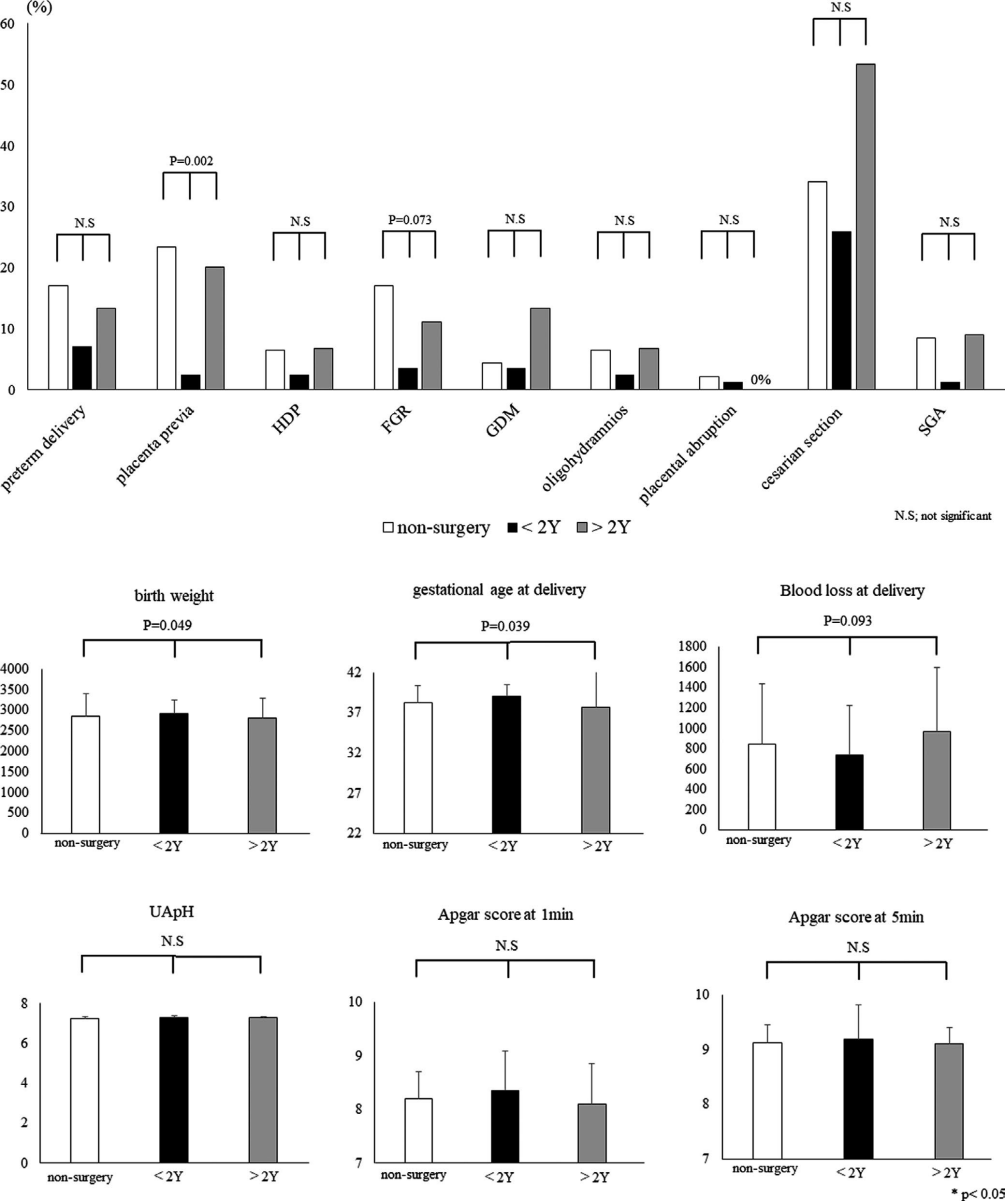
(A and B) Bar graphs illustrating perinatal outcomes in pregnant patients with endometriosis according to time from surgery to pregnancy (<2 years vs. >2 years) or non‐surgical history. Data are presented as mean ± standard deviation or as *n* (%). FGR, fetal growth restriction; GDM, gestational diabetes mellitus; HDP, hypertensive disorders of pregnancy; N.S., not significant; SGA, small for gestational age. *p*‐values < 0.05 are considered statistically significant

There were no differences in the other clinical backgrounds among the three groups. The recurrence rate of endometrioma was significantly higher in the >2Y group (24.0%, 12/50) than in the <2Y group (4.1%, 4/98; *p = *0.001, Table 3). The prevalence of placenta previa was lower in the <2Y group among the three groups (placenta previa; non‐surgery vs. <2Y vs. >2Y: 23.4%, 11/47 vs. 2.4%, 2/85 vs. 20.0%, 9/45: *p = *0.002) and the prevalence of FGR tended to be lower in <2Y group among the three groups (*p = *0.073) (Figure 2A). There were significant differences in the gestational age (38.3 ± 2.2 weeks vs. 39.1 ± 1.4 weeks vs. 37.7 ± 5.0 weeks: *p = *0.039) and birth weight (2858.8 ± 537.4 g vs. 2927.9 ± 310.7 g vs. 2813.0 ± 481.8 g: *p = *0.049) among non‐surgery, <2Y, and >2Y group; however, the prevalence of preterm delivery was not changed among three groups (*p = *0.335). There were no differences in the other perinatal outcomes among the three groups (Figure 2B, Supplemental Data 2).

**TABLE 3:** Maternal characteristics in pregnant patients with endometriosis according to time from surgery to pregnancy (<2 years vs. >2 years) or non‐surgical history

Pregnant cases with endometriosis (*N* = 208)	Non‐surgery (*N* = 60)^※1^	<2 years (*N* = 98)^※2^	>2 years (*N* = 50)^※3^	*p*‐Value^※1 vs.※2 vs ※3^
Age (years)	32.8 ± 4.9	32.1 ± 4.2	34.2 ± 4.8	0.289
Body mass index (kg/m^2^)	21.6 ± 3.4	21.7 ± 3.3	22.3 ± 3.4	0.477
Assisted reproductive technology	46.7% (28/60)	54.1% (53/98)	32.0% (16/50)	0.059
Parity	0.3 ± 0.6	0.3 ± 0.5	0.5 ± 0.6	0.113
Revised‐American Society for Reproductive Medicine (points)	‐	49.1 ± 31.8	69.3 ± 97.9	0.360^※2 vs. ※3^
Diameter of ovarian endometrioma (mm)	45.5 ± 16.4	46.8 ± 14.7	48.4 ± 16.1	0.450
Period from surgery to pregnancy (month)	‐	12.1 ± 7.3	58.8 ± 33.1	<0.001^※2 vs ※3^
Bilateral ovarian endometrioma	25.0% (15/60)	32.7% (32/98)	30.0% (15/50)	0.696
Leiomyoma	15.0% (9/60)	21.4% (21/98)	22.0% (11/50)	0.700
History of surgery for uterus	11.7% (7/60)	23.5% (23/98)	32.0% (16/50)	0.062
Miscarriage	21.7% (14/60)	13.3% (13/98)	10.0% (5/50)	0.195
Recurrence of endometrioma	‐	4.1% (4/98)	24.0% (12/50)	0.001^※2 vs ※3^

Note

Data are presented as mean ± standard deviation or as % (n/N). The analysis was performed by using Anova test and chi‐test by m×n contingency table. *p*‐values < 0.05 are considered statistically significant. ※1; Non‐surgery group, ※2; < 2 years group, ※3; > 2 years group.

To investigate which factors were associated with the prevalence risk of placenta previa, we performed a logistic regression analysis and found that history of laparoscopic surgery for endometriosis (odds ratio, OR: 0.32, 95% confidential interval, CI (0.11–0.90); *p = *0.032) was associated with a reduced risk of placenta previa. In the surgery group, multivariate analysis showed that pregnancy more than 2 years after surgery for endometriosis (OR: 7.98, 95% CI (1.51–61.63); *p = *0.014) was associated with an increased risk of placenta previa; however, postoperative recurrence of endometrioma (OR: 4.03, 95% CI (0.68–25.44); *p = *0.124) was not related to the risk of placenta previa. ART is known to be a factor that increases the prevalence of placenta previa;^20^ however, there was no association between ART and placenta previa in this study (Table 4).

**TABLE 4:** Logistic regression analysis of the factors related to the prevalence of placenta previa in the patients with endometriosis and the endometriosis patients with a history of surgery

Variable (the pregnant patients with endometriosis)	Odds ratio (95% CI)	*p*‐Value
Age >35 years	2.95 (1.09–8.29)	0.032
Body mass index >25.0 (kg/m^2^)	1.60 (0.39–5.45)	0.485
Assisted reproductive technology	1.38 (0.52–3.74)	0.512
History of pregnancy	1.57 (0.57–4.40)	0.380
Bilateral ovarian endometrioma	1.70 (0.60–4.68)	0.312
Leiomyoma	1.08 (0.19–6.60)	0.929
History of surgery for uterus	0.53 (0.07–2.81)	0.474
History of surgery for endometriosis	0.32 (0.11–0.90)	0.032

Variable (the endometriosis patients with a history of surgery)	Odds ratio (95% CI)	*p*‐Value
Age >35 years	1.57 (0.37–7.00)	0.538
Body mass index >25.0 (kg/m^2^)	2.43 (0.28–15.61)	0.384
Assisted reproductive technology	1.35 (0.30–5.90)	0.685
History of pregnancy	0.71 (0.16–2.85)	0.627
Bilateral ovarian endometrioma	1.64 (0.36–7.04)	0.507
Leiomyoma	1.61 (0.11–33.58)	0.727
history of surgery for uterus	0.867 (0.05–7.33)	0.906
Postoperative recurrence of endometrioma	4.03 (0.68–25.44)	0.124
Pregnancy more than two years from the surgery for endometriosis to pregnancy	7.98 (1.51–61.63)	0.014

Note

Data are presented as odds ratio (95% Confidential Interval). *P*‐values < 0.05 are considered statistically significant.

## 4 DISCUSSION

This study aimed to investigate the effect of laparoscopic surgery for endometriosis on perinatal outcomes in subsequent pregnancies. In recent years, much attention has been focused on the association between endometriosis and perinatal outcomes, such as miscarriage, placenta previa, preterm premature rupture of membranes, preterm birth, HDP, SGA, postpartum hemorrhage, placental abruption, stillbirth, and neonatal death.^8^, ^9^, ^10^, ^11^, ^12^, ^13^, ^15^, ^21^ Several reports have described the mechanism by which endometriosis may increase obstetric complications, demonstrating that chronic inflammation (e.g., cyclooxygenase‐2, interleukin‐8, adhesions, progesterone‐resistant endometrium, and vascularized environment due to endometriosis) could lead to various complications during pregnancy.^10^, ^22^, ^23^ Women with endometriosis may have altered uterine contractions, which may affect the location of blastocyst implantation, thereby increasing the risk of placenta previa.^24^, ^25^ Vercellini et al.^26^ pointed out that dense pelvic adhesions caused by endometriosis may inhibit the migration of the placenta away from the internal ostium of the uterus, leading to placenta previa. Maternal inflammation in patients with endometriosis may lead to deficient spiral artery remodeling and inadequate placenta formation, leading to preeclampsia and placenta‐related FGR.^27^ In the present study, the better perinatal outcomes in the surgery group may be due to the removal of these influential endometriotic lesions.

To date, although there are few papers on the impact of endometriosis surgery on perinatal outcomes, two papers have shown that surgery for endometriosis before pregnancy does not improve perinatal outcomes. Miura et al.^28^ reported that surgery before pregnancy did not decrease the prevalence of placenta previa, preterm birth, HDP, postpartum hemorrhage, GDM, and placental abruption. Using a national cohort in Denmark, Berlac et al.^29^ also showed that gynecological surgery for endometriosis before pregnancy did not improve perinatal outcomes. Contrary to their results, in the present study, a significant decrease in the prevalence of placenta previa was found in the surgery group compared with that of the non‐surgery group with endometriosis. In the previous papers, the authors pointed out the limitation that the time from surgery to pregnancy and the severity of endometriosis in the target patients were not clear.^28^, ^29^ These may have contributed to the difference between our results and theirs. A recent systematic review shows that severe endometriosis is associated with an increased prevalence of placenta previa, whereas non‐severe endometriosis was not.^17^ Therefore, in the present study, we determined the definition of endometriosis patients with endometriomas >3 cm in diameter, that is, only those with r‐ASRM classifications of stage III or IV. This is the strength point of this study that we focused on the cases with severe endometriosis. And then, we analyzed the influence of surgery, postoperative recurrence, and the time from surgery to pregnancy for obstetric outcomes. Previous reports demonstrate that the postoperative recurrence rate of endometriosis is relatively high, estimated to be 21.5% at 2 years and 40%–50% at 5 years, and is associated with the duration of postoperative follow‐up and the r‐ASRM stage at surgery.^30^, ^31^ When we diagnose a recurrence of endometriosis, identifying an ovarian endometrioma on imaging is considered the simplest method. On the contrary, when lesions recur in the pelvis, not the ovary, it is difficult to diagnose endometriosis by imaging. Therefore, in the present study, we evaluated the perinatal outcomes of subjects based on (1) recurrence of endometrioma and (2) timing after surgery (less or greater than 2 years). In pregnant women with no recurrence of ovarian endometrioma on ultrasonography, some perinatal risks such as placenta previa and FGR decreased. But, in the patients with recurrence, these perinatal risks returned to the same level as in the non‐surgery group. Conceiving within 2 years after surgery reduces several perinatal risks, but these risks return with a lapse of >2 years. It is noteworthy that the prevalence risk of placenta previa is significantly reduced both in the pregnant patients with postoperative non‐recurrence and those conceiving within 2 years after surgery, but the prevalence risk of placenta previa returns to the preoperative level in cases with recurrence or >2 years from surgery to pregnancy. Another strength point is that the surgery for endometriosis was found to be associated with a reduction in placenta previa by the multivariate analysis in several risk factors for the prevalence of placenta previa including ART. These may suggest that surgery for endometriosis reduces pelvic inflammation and decreases the prevalence risk of placenta previa, but over time, endometriosis flares up and increases inflammation, again affecting perinatal outcomes. Despite of the high prevalence of placenta previa (28.6%) in cases of recurrent endometrioma, multivariate analysis showed that recurrence of chocolate cysts were not related ṭo an increased risk of placenta previa (*p = *0.124). This may be due to the small number of recurrent cases (14 cases), and further study is needed. From the results of the present study, it may be better to aim for early pregnancy after surgery to reduce obstetric complications, especially placenta previa.

This study has several limitations. First, in the non‐surgery group, endometriosis was diagnosed based on ultrasonography and/or MRI, which are less reliable than the reference standard laparoscopy. Second, although the size of ovarian endometrioma was not different between the surgery and non‐surgery groups, there may be a difference in the severity of endometriosis between the two groups, since pelvic endometriosis cannot be assessed by imaging, and this may affect the prevalence of placenta previa. Third, this was a retrospective study, and the sample size was smaller than those in previous studies.^28^, ^29^ Fourth, as the medical treatments for endometriosis, especially hormonal treatments, have changed over the 15‐year period, we cannot deny that these effects may have affected the patient outcome. Fifth, since we could not examine how the intra‐peritoneal environment and general condition of the patient changed postoperatively, the mechanism of improvement in perinatal prognosis is not fully understood.

In conclusion, laparoscopic surgery for endometriosis may decrease the prevalence of placenta previa in the subsequent pregnancy. However, since the prevalence risk of placenta previa may be re‐increased if more than two years have passed from the surgery to pregnancy, it may be better to recommend to conceive within two years. Further prospective studies are needed in the future.

## DISCLOSURES

We have no conflict of interest to disclose. This study was approved by the hospital's ethics committee and has been approved by the IRB. We are in compliance with the Statement of Human Rights. Because this is a non‐invasive retrospective study, instead of obtaining informed consent from each patient, we are disclosing information about this study on the institution's website. In addition, this study does not include any animal experiments. This study is a retrospective observation of patients who have already completed standard treatment and have not been enrolled in any clinical trial registry.

## Supporting information

Supplementary Material

## Data Availability

None.
