# Supplementary material for: Influence of laparoscopic surgery for endometriosis and its recurrence on perinatal outcomes
Source: Reprod Med Biol. 2022 Apr 6;21(1):e12456. doi: 10.1002/rmb2.12456 (PMC8986974; doi:10.1002/rmb2.12456)
Supplement: Supplementary file 1 — Supplementary Material [file RMB2-21-e12456-s001.docx]

Supplemental Data 1 Comparison of perinatal outcomes between pregnant patients with endometriosis without surgery, with postoperative *non-recurrence* of endometrioma, and with postoperative *recurrence* of endometrioma

| Pregnant cases with endometriosis（N=177） | *non-surgery* (N=47）^※1^ | *non-recurrence* （N=116）^※2^ | *recurrence* (N=14）^※3^ | P-value  ^※1 vs ※2 vs ※3^ |
| --- | --- | --- | --- | --- |
| preterm delivery | 17.0% (8/47) | 9.5% (11/116) | 7.1% (1/14) | 0.537 |
| placenta previa | 23.4% (11/47) | 6.0% (7/116) | 28.6% (4/14) | 0.007 |
| hypertensive disorders of pregnancy | 6.4% (3/47) | 4.3% (5/116) | 0% (0/14) | 0.946 |
| fetal growth restriction | 17.0% (8/47) | 4.3% (5/116) | 21.7% (3/14) | 0.048 |
| gestational diabetes mellitus | 4.3% (2/47) | 9.5% (9/116) | 0% (0/14) | 0.787 |
| oligohydramnios | 6.4% (3/47) | 3.4% (4/116) | 6.3% (1/14) | 0.900 |
| placental abruption | 2.1% (1/47) | 0.9% (1/116) | 0% (0/14) | 0.678 |
| cesarean section | 34.0% (16/47) | 32.8% (38/116) | 57.1% (8/14) | 0.319 |
| gestational age at delivery (weeks) | 38.3±2.2 | 38.6±3.3 | 38.4±2.0 | 0.771 |
| birth weight (g) | 2858.8±537.4 | 2899.6±515.0 | 2791.4±448.7 | 0.621 |
| small-for-gestational age | 8.5% (4/47) | 3.4% (4/116) | 21.7% (3/14) | 0.107 |
| umbilical artery pH | 7.26±0.06 | 7.28±0.06 | 7.23±0.1 | 0.043 |
| the blood loss at delivery (g) | 845.7±587.4 | 785.0±515.0 | 1086.4±769.7 | 0.180 |
| Apgar score (1 min) | 8.2±0.5 | 8.3±0.8 | 8.3±0.5 | 0.890 |
| Apgar score (5 min) | 9.1±0.3 | 9.1±0.5 | 9.2±0.4 | 0.908 |

Data are presented as mean ± standard deviation or as n (%).

NS: not significant. Statistical significance was set at P < 0.05.

Supplemental Data 2 Comparison of perinatal outcomes between pregnant patients with endometriosis according to time from surgery to pregnancy (*< 2 years* vs. > *2 years*) or non-surgical history

| pregnant cases with endometriosis（N=177） | *non-surgery* (N=47）^※1^ | *< 2 years* (N=85）^※2^ | *> 2 years*（N=45）^※3^ | P-value  ^※1 vs ※2 vs ※3^ |
| --- | --- | --- | --- | --- |
| preterm delivery | 17.0% (8/47) | 7.1% (6/85) | 13.3% (6/45) | 0.335 |
| placenta previa | 23.4% (11/47) | 2.4% (2/85) | 20.0% (9/45) | 0.002 |
| hypertensive disorders of pregnancy | 6.4% (3/47) | 2.4% (2/85) | 6.7% (3/45) | 0.715 |
| fetal growth restriction | 17.0% (8/47) | 3.5% (3/85) | 11.1% (5/45) | 0.073 |
| gestational diabetes mellitus | 4.3% (2/47) | 3.5% (3/85) | 13.3% (6/45) | 0.698 |
| oligohydramnios | 6.4% (3/47) | 2.4% (2/85) | 6.7% (3/45) | 0.756 |
| placental abruption | 2.1% (1/47) | 1.2% (1/85) | 0% (0/45) | 0.894 |
| cesarean section | 34.0% (16/47) | 25.9% (22/85) | 53.3% (24/45) | 0.255 |
| gestational age at delivery (weeks) | 38.3±2.2 | 39.1±1.4 | 37.7±5.0 | 0.039 |
| birth weight (g) | 2858.8±537.4 | 2927.9±310.7 | 2813.0±481.8 | 0.049 |
| small-for-gestational age | 8.5% (4/47) | 1.2% (1/85) | 8.9% (4/45) | 0.289 |
| umbilical artery pH | 7.26±0.06 | 7.28±0.08 | 7.27±0.06 | 0.365 |
| the blood loss at delivery (g) | 845.7±587.4 | 736.0±484.5 | 963.8±636.3 | 0.093 |
| Apgar score (1 min) | 8.2±0.5 | 8.4±0.7 | 8.1±0.8 | 0.217 |
| Apgar score (5 min) | 9.1±0.3 | 9.2±0.6 | 9.1±0.3 | 0.779 |

Data are presented as mean ± standard deviation or as n (%).

NS: not significant. Statistical significance was set at P < 0.05.
